# Tumor Apolipoprotein E is a key checkpoint blocking anti-tumor immunity in mouse melanoma

**DOI:** 10.3389/fimmu.2022.991790

**Published:** 2022-10-19

**Authors:** Xiaofang Wu, Priya Srinivasan, Mousumi Basu, Peng Zhang, Michele Saruwatari, Bernice Thommandru, Ashley Jacobi, Mark Behlke, Anthony Sandler

**Affiliations:** ^1^ The Joseph E. Robert Jr. Center for Surgical Care and The Sheikh Zayed Institute for Pediatric Surgical Innovation, Children’s National Hospital, George Washington University, Washington, DC, United States; ^2^ Beijing Key Laboratory for Genetics of Birth Defects, Beijing Pediatric Research Institute, Beijing Children’s Hospital, Capital Medical University, National Center for Children’s Health, Beijing, China; ^3^ Integrated DNA Technologies, Inc., Coralville, IA, United States

**Keywords:** apoE, immune-checkpoint, melanoma, T cells, dendritic cells

## Abstract

Immunotherapy is a key modality in the treatment of cancer, but many tumors remain immune resistant. The classic mouse model of B16-F10 melanoma is immune resistant even in the face of checkpoint inhibition. Apolipoprotein E (apoE), a known immune suppressant is strikingly elevated in many human tumors, but its role in cancer immunology is not defined. We investigated the role of apoE in the immune micro-environment using a mouse melanoma model. We demonstrate that ApoE is -highly expressed in wild-type B16-F10 melanoma and serum levels progressively increase as tumors grow. The conditioned media from wild type ApoE secreting melanoma cells suppress T-cell activation *in vitro* while this suppressive effect is absent in conditioned media from ApoE knock out tumor cells. Mechanistically, apoE induces IL-10 secreting dendritic cells and stimulates T-cell apoptosis and arrest partially *via* the lrp8 receptor. Ablating ApoE in mice inoculated with tumor cells enabled tumor cell rejection and was associated with induction of immune pathway activation and immune cell infiltration. Tumor secreted apoE appears to be a potent immune cell checkpoint and targeting apoE is associated with enhanced tumor immunity in the mouse melanoma model.

## Introduction

The need for more effective therapy of tumors is evident in the poor outcomes of high-risk or advanced disease. Cancer vaccines and immune-based therapies hold great promise for malignant solid tumors, but despite robust immune activation with targeted checkpoint inhibitors, cure is often elusive. Immune based therapies (and specifically tumor vaccines) are frequently constrained by intrinsic tumor cell mechanisms enabling immune privilege/evasion. We identify tumor secreted apoE as a novel checkpoint enabling immune evasion in a mouse melanoma tumor model.

ApoE is a polymorphic multifunctional protein, classically considered to play a critical role in atherosclerosis and neurodegenerative diseases (1). Knockout mice fed an atherogenic diet develop pronounced hypercholesterolemia along with an immune-activated phenotype (2, 3). Experimental models reveal a critical function for myeloid derived apoE modulating DC antigen presentation and T-cell priming (2). ApoE attenuates inflammation by complex formation with activated C1q (4), while most recently it was shown that common germline variants of the human APOE gene modulate melanoma progression and survival (5).

In tumors, APOE itself is shown to act as an autocrine or paracrine modulator of carcinogenesis (6, 7). In several human cancers, APOE gene expression is significantly higher in cancer tissue than in adjacent non-cancer tissue (8–13) and higher levels of tumor APOE are associated with metastasis (14). In pancreatic ductal adenocarcinoma (PDA) patients, elevated plasma APOE protein levels are associated with poor survival, whereas tumor associated macrophages that are key drivers of immunosuppression are characterized by elevated levels of ApoE in both mouse and human PDA (15). Further studies reveal that apoE KO mice have less orthotopic mammary tumor development and pulmonary metastasis than wild type (WT) mice (16) and lung tumor development and metastasis are suppressed through enhancing anti-tumor activity of natural killer (NK) cells (6). In contrast, it is reported that apoE is involved in the inhibition of melanoma metastases and has anti-angiogenic properties (17). ApoE promoted anti-tumor immunity by targeting infiltrating innate myeloid derived suppressor cells (MDSC) *via* Liver X receptor (LXR) agonism (18). Furthermore, pretreating cancer cells with apoE inhibited their growth in mouse models. These conflicting observations suggest that the context in which apoE is engaged and the specific APOE genotype in humans may determine its effects, but apoE appears to inhibit its cellular target in most circumstances. The known association of apoE with immunosuppression and its conflicting observations described in tumor biology led us to examine the role of apoE in an immune resistant mouse melanoma model.

We investigated the expression of apoE in mouse melanoma and the role of apoE in the context of tumor growth and immunity. ApoE is abundantly present in B16-F10 melanoma tumors and serum levels of apoE increase dramatically with melanoma tumor growth. Mechanistically, apoE induces IL-10 secreting suppressive dendritic cells and directly inhibits T-cell function at least partially *via* the lrp8 receptor. Ablating ApoE in mouse melanoma enabled tumor cell rejection and induced robust immune activation and tumor immunity. The results reveal a critical role for tumor secreted apoE as a comprehensive checkpoint which appears to alter dendritic cell function and inhibit T-cell efficacy. ApoE is a novel checkpoint with extensive and potent suppressant effects in mouse melanoma. It is anticipated that targeting apoE will augment immune based therapy in apoE secreting immune resistant tumors.

## Materials and methods

### Animals

Female C57BL/6 mice and apoE**^-/-^** mice (B6.129P2-Apoe^tm1Unc/J^) aged 6 weeks were purchased from Jackson Laboratories (Bar Harbor, Maine, United States). Lrp8**^-/-^** C57BL/6 breeder mice were generously provided by Dr. Sohail Tavazoie’s laboratory from the Rockefeller University. Mice were housed five per cages and kept in a temperature-controlled environment (20 ± 2°C, 50 ± 5% relative humidity) with a 12-hour light/dark cycle in an air-conditioned room with free access to food and water. The animals were acclimated for 4–5 days prior to tumor challenge. All procedures were approved by the Institutional Animal Care and Use Committee (IACUC) of Children’s National Hospital, Washington, DC.

### Cells

The murine melanoma B16-F10 cell line (purchased from ATCC^®^ CRL-6475, VA), were cultured in DMEM (Life Technologies, CA) supplemented with 10% heat-inactivated FBS (Sigma-Aldrich) and 100 IU/ml Penicillin, 100 μg/ml Streptomycin (Life Technologies).

### Generation of apoE^-/-^ cell lines *via* CRISPR genome editing in B16-F10 cells

Six guide RNAs (gRNAs) were designed using IDT’s gRNA design tool, targeting the mouse apoE gene. gRNAs were synthesized as 2-part crRNA: tracrRNA gRNAs with chemical modifications (Integrated DNA Technologies, Inc., IA) and were functionally screened by next generation sequencing (NGS) for high INDEL (insertion/deletion) frequency and low in-frame INDEL rates. gRNAs were prepared by complexing a 1:1 molar ratio of the crRNA: tracrRNA at a final concentration of 100 μM, heating to 95°C and slowly cooling to room temperature. Ribonucleoprotein complexes (RNPs) were formed by the addition of purified Alt-R HiFi Cas9 protein (IDT) to each gRNA at a 1.2:1 molar ratio in 1× PBS to a concentration of 5.6 μM. RNP complexes were allowed to form for 10 min at room temperature before electroporation. RNP complexes (5 μL), Alt-R Cas9 Electroporation Enhancer (3 μL), and 350,000 B16-F10 cells (20 μL) resuspended in Buffer SF were mixed and electroporated using the Lonza 96-well Shuttle System (Lonza, Basel, Switzerland) with electroporation protocol 96-DS-150. Final concentrations for RNP and Alt-R Cas9 Electroporation Enhancer were 1 μM and 4 μM, respectively. Genomic DNA was extracted 48 hours post-transfection using QuickExtract DNA extraction solution (Epicentre Biotechnologies, CA) according to the manufacturer’s specifications. The targeting sequence for each ApoE crRNAs are listed in **Supplementary Table 1**.

### Isolation of monocolonal apoE knockouts

The lead gRNA (Mm. Cas9.APOE.1-E) resulted in a 99% INDEL frequency with no in-frame INDELs and was electroporated into B16-F10 cells using the Lonza 96-well Shuttle System as previously described. The electroporated cells were plated in 1 well of a 6-well plate and allowed to grow until confluent. The cells were then dissociated by trypsinization, resuspended in media, and counted. The suspension was diluted to 20,000 cells/mL; 4000 cells were added to 1 well of a 96-well plate and then diluted by array dilution. After 5 days of growth, each well was visually screened for single colonies. Wells with only 1 colony were allowed to grow to confluency. Each well was progressively passaged to a larger well until confluent in a 100 mm dish, about 8.8 x 10^6 cells for genomic DNA extraction and further cell passaging. The genomic DNA from each well was subject to quantification of total editing and analysis of INDEL profile by NGS to confirm a monoclonal isolate.

### Quantification of total editing and analysis of INDEL profiles by rhAmpSeq

Genomic DNA libraries for sequencing were prepared using IDT rhAmpSeq targeted amplification. In short, the first round of PCR was performed using target-specific primers with universal 5’ tails (**Supplementary Table 2**); a second round of PCR incorporated P5 and P7 Illumina adapter sequences to the amplicon ends. Libraries were purified using Agencourt^®^ AMPure^®^ XP system (Beckman Coulter, Brea, CA, USA) with a 1:1 ratio of beads to reaction by volume and quantified with quantitative real-time PCR (qPCR) before loading onto the Illumina^®^ MiSeq platform (Illumina, San Diego, CA, USA). Paired end 150 base pair reads were sequenced using V2 chemistry. A sequencing depth of at least 1000 reads was obtained for each sample. Total editing efficiency was calculated and INDEL profiles were evaluated using an IDT custom-built pipeline, CRISPAltRations.

### Antibodies and reagents

Anti (α)-mouse CTLA-4, and mouse IgG2b isotype antibodies were purchased from BioXCell (West Lebanon, NH). COG133 and JQ1 were purchased from Tocris (Minneapolis, MN). Dynabeads™ Mouse T-Activator CD3/CD28 for T-Cell Expansion and Activation kit, Vybrant™ DyeCycle™ Violet Stain kit, SYTOX™ red dead cell stain kit, CellTrace™ far red cell proliferation kit, Live/Dead fixable aqua dead cell stain kit, Brilliant stain buffer and mouse IL-2 Carrier-Free recombinant protein were purchased from Thermo Fisher (Waltham, MA).

### Multiplex cytokine/chemokine analysis

Cell culture supernatant was collected after centrifuge at 1,200 rpm for 10 min at 4°C. The concentrations of the following immune molecules were determined using the mouse Cytokine & Chemokine 36-plex ProcartaPlex Panel, a magnetic bead-based multiplex immunoassay (Thermo Scientific) following our previous protocol (19). Briefly, cell culture supernatant samples were mixed with antibody-linked polystyrene beads on a 96-well plate and incubated at room temperature (RT) for 2 h on an orbital shaker at 500 rpm. After washing, plates were incubated with biotinylated detection antibody for 30 min at RT. Plates were then washed twice and then labeled beads were re-suspended in streptavidin-PE. Each sample was measured in duplicate along with standards (8-point dilutions) and the buffer control. Plates were read using a Luminex Bio-plex 200 system (Bio-Rad Corp.) for quantitative analysis.

### Isolating T cells from mouse spleen

Spleens were collected from mice euthanized by CO_2_ narcosis and cervical dislocation. Spleens were pulverized through a 40-μm mesh cell strainer and treated with ACK lysing buffer (Thermo Fisher) for 10 seconds to remove erythrocytes. According to the manufacture’s instruction of the Pan T Cell Isolation Kit (Miltenyi Biotec), 10 µL of Pan T Cell Biotin Antibody Cocktail was added per 10^7^ splenocytes and incubated for 5 min at 4°C. Subsequently, 20 µL of Pan T Cell MicroBead Cocktail was added per 10^7^ cells. Following incubation for 10 min at 4°C, the mixed cell suspension was applied onto the LS column (Miltenyi Biotec). The flow-through containing unlabeled cells, representing the enriched T cells were collected. T cells were cultured in RPMI 1640 media containing 30 U/mL IL-2 (Thermo Fisher) and stimulated with Dynabeads^®^ Mouse T-Activator CD3/CD28 magnetic beads (Thermo Fisher) at a 1:1 ratio (cell: bead).

### IFNγ measurement

WT and apoE^-/-^ mice or WT and lrp8^-/-^ mice, were inoculated with either WT B16 or apoE^-/-^ B16 cells, with anti-CTLA4 antibody on day 0. These vaccinated splenocytes (VS) were harvested on day 7 and co-cultured with either WT B16 or apoE^-/-^ B16 cells for 48hr, following which IFNγ levels in media were compared with ELISA assay. To set up co-culture, a total of 5 × 10^5^ freshly isolated mouse T cells or splenocytes were plated in a volume of 600 μl per well of 24-well plates, then they were co-cultured with 5 × 10^4^ B16 cells and stimulated with or without CD3/CD28 Dynabeads. Cells were exposed to apoE agonist COG133 at 0, 0.3, 3, 9, 15, 30 µM or human anti-APOE antibody at 1 µg/ml, 10 µg/ml and 20 µg/ml at 37°C under 5% CO2 for 24 hr or 48 hr. Supernatants were collected from triplicate wells, and IFNγ was assayed using the mouse uncoated IFNγ ELISA kit from Invitrogen (Carlsbad, CA). Readings were measured at 450 nm using the EnSpire 2300 Multilabel plate reader (Perkin Elmer, Waltham, Massachusetts, US).

### Cell lines and conditioned media

5x10^6^ wild type (WT) and apoE^-/-^ B16 cells were irradiated at 60 Gy and then cultured in 20 mL DMEM media supplemented with 10% FBS for 48 hr in T75 flask. The conditioned media (CM) was collected and centrifuged at 1100 rpm at room temperature for 5 min. The supernatant was aliquoted and stored at -80°C.

### Cell cycle assay

For testing cell cycle, 1x10^6^ T cells in 1 mL DMEM complete media were incubated with 5 µM Vybrant™ DyeCycle™ Violet Stain (Thermo Fisher) at 37˚C for 30 min. And then 5 µl 7AAD were added and incubated for 10 min prior to analysis. Total 25,000 cells were analyzed per measurement. The same forward and side scatter gates were applied to each sample, and within that gate we measured the intensity of vibrant cell cycle dye. Samples were analyzed on a flow cytometer using 405 nm excitation and 440 nm emission. To compare the growth rates of B16 WT and apoE^-/-^ cells, the Click-iT Edu Alexa Fluor 488 flow cytometry assay kit was used in conjunction with the FxCycle Violet stain from Invitrogen (Carlsbad, CA). 50,000 cells were plated per well of a 6 well-plate and cell cycle was analyzed at 48 hr as per the manufacturer’s directions.

### Measurement of apoE in serum of mice

Mouse serum was collected from naïve WT C57/BL6 mice and also from both naïve and tumor-bearing C57/BL6 apoE^-/-^ mice. ApoE levels in mouse sera were quantified using the mouse apoE ELISA^PRO^ kit from Mabtech (Cincinnati, OH) as per the manufacturer’s directions.

### Nanostring

RNA was extracted and gene expression was directly measured *via* counts of corresponding mRNA in each sample using an nCounter murine PanCancer Immune Profiling Panel (NanoString, Seattle, WA, USA). For full details, see our previous publication (19). Briefly, 100 ng of high-quality total RNA were hybridized with reporter probes, and then biotinylated capture probes at 65°C for 16–18 hr before being placed into the nCounter Prep station in which samples were affixed to a cartridge. Cartridges were then read by the nCounter Digital Analyzer optical scanner. Further advanced immune-profiling analysis was performed using nSolver 4.0 analysis software with nCounter advanced analysis package (NanoString Technologies) with identified immune cell types. Genes were grouped into 14 immune cell types and 39 immune functions according to the manufacturer’s designation (19).

### Quantitative real-time RT-PCR

Quantitative real-time PCR (qPCR) was performed using TaqMan^®^ Gene Expression Master Mix (Life Technologies) in a QuantStudio 7 Flex Real-Time PCR System (Thermo Fisher Scientific, Waltham, MA) following the methods that we published previously (19). Each reaction was performed in triplicate, including no template controls and amplification of a housekeeping gene, GAPDH. Gene-specific assays were Mm01307192_m1 for apoE, Mm00464608_m1 for Lrp1, Mm01328171_m1 for Lrp2, Mm00474030_m1 for Lrp8, Mm01177349_m1 for Ldlr, Mm00443298_m1 for Vldlr, Mm99999915_g1 for Gapdh (Life Technologies, Thermo Fisher). Changes in relative gene expression normalized to GAPDH levels were determined using the ΔΔCt method. Results were averaged and statistically analyzed using t-tests.

### Mouse melanoma models

C57BL/6 wild type (WT) mice, C57/BL6 apoE knockout (apoE^-/-^) mice, and C57/BL6 lrp8 knockout (lrp8^-/-^) mice were injected subcutaneously in the right flank with 1 × 10^4^ freshly prepared B16 WT or apoE^-/-^ tumor cells in 100 µl 1xPBS on day 0 and euthanized once the tumor reached 20mm in any dimension. Tumor growth was recorded every day by measuring the diameter in 2 dimensions using a caliper when appropriate as we have previously published (19, 20). Briefly, tumor volume was calculated using the following formula: large diameter^2^ × small diameter × 0.52. A tumor size of 20 mm in diameter in any dimension was designated as the endpoint, and mice were euthanized at that time. Euthanasia was achieved through cervical dislocation after CO2 narcosis. If a tumor impaired the mobility of an animal, became ulcerated, or appeared infected, or a mouse displayed signs of “sick mouse posture”, the mouse was euthanized. All the procedures are approved by the IACUC at CNMC and are in accordance with the humane care of research animals.

### Characterization of mouse tumors by immunohistochemistry (IHC)

Tumor was fixed in 10% neutral buffered formalin (pH 6.8–7.2; Richard-Allan Scientific, Kalamazoo, Michigan, US) for paraffin embedding and sectioning. Five μm tissue sections were cut with a microtome, and sample processing and IHC staining were performed as previously described (19) using rabbit polyclonal to CD45 and CD3 antibodies (1:200. Abcam, Cambridge, Massachusetts, US). Isotype-matched antibodies were used for negative controls.

### Statistical analysis

Statistical analysis of nanostring gene expression, normalization, clustering, Pathview plots and fold-changes were performed using the Advanced Analysis Module in the nSolver™ Analysis Software version 4.0 from NanoString Technologies (NanoString Technologies, WA, USA) following our published methods (19). Briefly, raw data for each sample were normalized to the geometric mean of housekeeping genes using the geNorm algorithm. Pathway scores were calculated as the first principal component (PC) of the pathway genes’ normalized expression. Each cell type score has been centered to have mean 0 and as abundance estimates (cell type scores) are calculated in log2 scale, an increase of 1 corresponds to a doubling in abundance. All differentially expressed genes were subjected to KEGG term analysis, with significance accepted at p < 0.05. The Benjamini-Yekutieli method was used to control the false discovery rate. All statistical analyses of nanostring data were carried out in R v3.4.3 software.

Statistical significance for each set of experiments was determined by the unpaired 2-tailed Student’s t-test, and the specific tests were indicated in the figure legends. The data are expressed as the mean ( ± SD), with p<0.05 considered statistically significant.

### Human melanoma RNA-seq analysis

We accessed the raw RNA-seq data of melanoma biopsies through the Gene Expression Omnibus database (accession number: GSE78220) (21) - Raw reads mapping to the reference genome (GRCh38) were performed on quality-checked and trimmed reads using STAR 2.4.1c (22). The reference annotation is Ensembl v86. The overlap of reads with annotation features found in the reference.gtf were calculated using HT-seq v0.6.1 (23). - The output computed for each sample (raw read counts) was then used as input for DESeq2 (24) analysis. Raw counts were normalized using DESeq2 function “rlog,” and normalized counts were used for downstream analysis. Statistical calculations were performed using GraphPad Prism software (GraphPad Software, San Diego, CA, USA) or R Software (Version 4.0).

## Results

### ApoE is highly expressed in the melanoma B16-F10 cell lines and apoE serum levels rise with tumor growth *in vivo*

**Figure 1:**
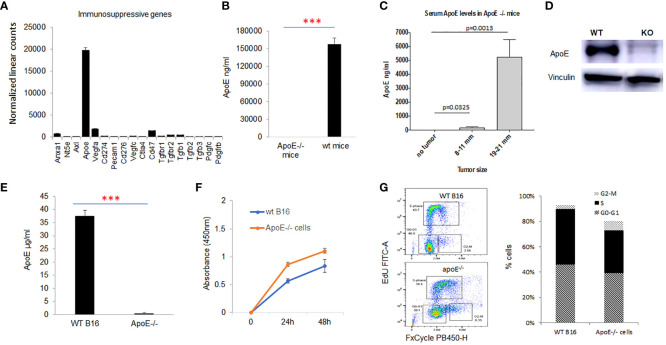
ApoE is the most highly expressed immune suppressive gene in the melanoma B16-F10 cell lines and apoE serum levels rise with tumor growth in vivo. **(A)** mRNA expression as revealed by nanostring nsolver Pancancer immune profiling, of the immune suppressive marker genes in B16F10 cells (n=6). **(B)** ApoE expression was undetectable in the serum of apoE knockout (apoE-/-) mice, but present at high levels in wild type (C57/BL6) mice with ELISA assay. **(C)** ApoE knockout mice were inoculated with 10^4 wild type (B16F10) cells and serum levels of apoE increased over time with increasing tumor size. **(D)** Validation of apoE gene knockout in B16F10 cells by CRISPR-Cas9 gene deletion. The level of apoE protein expression was measured in WT B16F10 and the corresponding apoE-/- clone by Western Blot. **(E)** Equivalent numbers of WT and apoE-/- B16 cells were plated and apoE levels released into culture media were quantified by ELISA at 48hr. ApoE is secreted at high levels in WT B16 cells and is not detectable in the apoE-/- cell line. **(F)** To evaluate whether targeting apoE influenced cell viability and proliferation, 5x10^4 WT (n=6) or apoE-/- B16 cells (n=6) were grown in 12-well plates and proliferation rates were measured at 24hr and 48hr by MTT assay. There is no statistically significant difference in cell proliferation rate between the two groups. **(G)** Cell cycle distribution was determined in WT and apoE-/- B16 cells. The various phases of the cell cycle are differentiated in the flow cytometry plot on the left: G0-G1 is the pre-synthesis phase, S-phase cells are undergoing active DNA synthesis and G2-M cells are preparing for mitosis. Bar graphs represents the percentage of cells in G0-G1, S and M phase of the cell cycle. Cell counts and cell cycle distribution indicate that WT and apoE-/- B16 cells proliferate at equivalent rates. Data are representative of three independent experiments. Results are expressed as mean score ±SD. ***p<0.001, determined by unpaired two-tailed Student’s t-test.

RNA was extracted and gene expression was directly measured *via* counts of corresponding mRNA in B16-F10 cells using an nCounter murine PanCancer Immune Profiling Panel (NanoString, Seattle, WA, USA). We evaluated the presence of multiple genes associated with immunosuppression in the B16-F10 melanoma cell line, ApoE was the most highly expressed immune-suppressive transcript in the cell line. (**Figure 1A**) ApoE is also constitutively detected in the serum of wild type (WT) C57/BL6 mice at high levels (**Figure 1B**). To evaluate the systemic levels secreted from the mouse melanoma tumor itself, we measured the level of serum apoE in apoE KO (apoE^−/−^) C57/BL6 mice inoculated subcutaneously (s.c.) with 10^4^ WT B16 (F10) cells injected in the right thigh. Blood and tumors were collected at various sizes until the tumors reached a max of 21 mm in any dimension. The serum levels of apoE in these tumor-baring apoE^-/-^ mice increased progressively and considerably with tumor growth (**Figure 1C**).

To further investigate the function of apoE, we generated apoE^-/-^ B16-F10 cell lines with CRISPR-Cas9 gene deletion. To confirm suppression or deletion of apoE protein, we performed western blot analysis on total protein lysates from B16 WT and apoE^-/-^ single clones. Our data shows absent apoE expression in the apoE^-/-^ single clones (**Figure 1D**). The apoE protein secreted from WT B16 and apoE knockout clones in culture media was quantified by ELISA assay and levels correlated with the protein expression pattern of western blot analysis confirming KO of apoE in the KO cell lines. (**Figure 1E**)

We then evaluated the *in vitro* proliferation rate (**Figure 1F**) and cell cycling (**Figure 1G**) of the WT and KO cell lines to determine if targeting apoE influenced cell viability and proliferation. There is no statistically significant difference between WT and ApoE^-/-^ cells in their proliferation rate or cell cycles (**Figures 1F, G**).

### ApoE secreted into media from B16 melanoma tumor cells inhibits T-cell function

**Figure 2:**
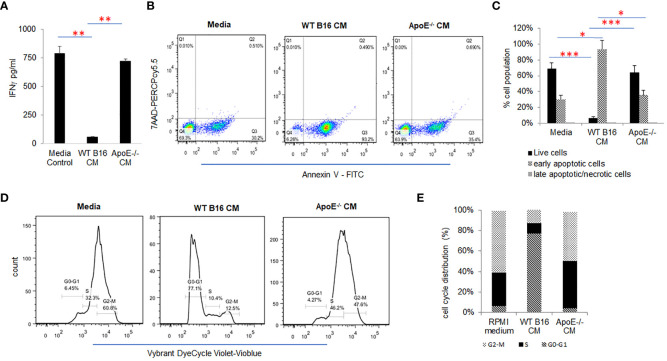
ApoE secreted into media from B16 melanoma tumor cells inhibits T-cell proliferation and function. **(A)** Conditioned media (CM) from WT or apoE^-/-^ cells was cultured with T-cells activated by CD3/CD28 beads. Bar graphs depict IFNγ released under these conditions. The production of IFNγ was significantly suppressed when T cells were cultured in WT B16 CM, but this suppression was reversed in apoE free CM from apoE^-/-^ cells. **(B)** Representative flow cytometry plots and **(C)** quantification of T cell apoptosis in CM. Results show that T cell viability is markedly reduced in the WT CM, whereas induction of apoptosis was reversed when cells were cultured in the apoE^-/-^ medium, similar to RPMI control medium alone. **(D)** Representative flow cytometry plots and **(E)** quantification of cell cycle analysis of T cells in different media. Cell cycle distribution showed an arrest of activated T cells in the G0-G1 phase in the WT B16 medium, while in the apoE^-/-^ medium most T cells were distributed in the S and G2-M phases, similar to the RPMI media control. Results are expressed as mean score ± SD. *p<0.05; **p<0.005; ***p<0.001, determined by unpaired two-tailed Student’s t-test.

To investigate the effect of tumor cell secreted apoE on activated T cells, splenic derived C57/BL6 T cells were cultured in control media and melanoma B16 WT or apoE**^-/-^** conditioned media (CM) in which the T cells were activated with CD3/CD28 beads for 48 hr. Cytokine secretion, apoptosis and proliferation of the cultured mouse T cells were examined and compared to T cells cultured in the RPMI media that served as controls (**Figure 2**). IFNγ production (**Figure 2A**) and T cell viability (**Figures 2B, C**) were significantly (P < 0.05) suppressed when T cells were cultured in WT B16 CM at 48 hr of incubation as compared to the RPMI media control, whereas apoE^-/-^ conditioned media was similar to RPMI control media alone and did not inhibit T cell activity nor viability (**Figures 2A–C**). In addition, cell cycle distribution showed that WT conditioned media arrested activated T cells in the G0/G1 phase while apoE**^-/-^** conditioned media-maintained T-cells in the S and G2M phase of the cell cycle, similar to control media without prior exposure to tumor cells. (**Figures 2D, E**). These results indicate that conditioned media derived from B16 tumor cells induces arrest of stimulated T cells in the G0/G1 phase of the cell cycle, resulting in apoptosis and suppression of cytokine production. In contrast, the singular absence of apoE in conditioned media rescues the activated T cell phenotype when stimulated with CD3/CD28 beads. To further define the cytokine response, we used a ProcartaPlex multiplex immunoassay, and quantified multiple cytokines/chemokines in the stimulated T-cell media from the same experiment. Ten out of 27 detectable cytokines suppressed by WT conditioned media returned to baseline levels or were upregulated when T cells were cultured in apoE**^-/-^** CM. These included effectors: IFNγ, IFNα, TNFα; stimulator, IL18; inflammatory factor: IL4, IL13; chemo attractive factor: MCP-1, MCP3, MIP-1α, MIP-1β; and regulatory factor: IL-10 (P < 0.05) cytokines. IL6, CCL-5 (Rantes) and GroαKC were downregulated (**Supplement Figure 1A**).

To further delineate the T-cell suppressive effect directly, COG133, a fragment of apoE peptide, which competes with the apoE holoprotein for binding the low-density lipoprotein (LDL) receptors and acts as an apoE mimetic, was tested to determine its effect on T cell activation. T cells were activated with CD3/CD28 dynabeads and the effects of COG133 (0, 3, 9, 15 and 30µM concentrations) on T cell apoptosis were evaluated. The T cell viability was diminished in a dose dependent manner (**Supplement Figure 1B**). These results confirm the immunomodulatory role of apoE on activation of T cells showing robust and extensive suppression of T cell function.

### ApoE secreted from B16 melanoma tumor cells in culture may also impair activation of pro-inflammatory dendritic cells

Activation of innate antigen presenting cells like dendritic cells (DC) are critical for effective induction of immunity. We tested the effect of conditioned media on toll like receptor (TLR7/8) stimulated primary bone marrow derived dendritic cell activation. B16 WT conditioned media (CM) suppressed DC activation as determined by cytokine production, but this effect was absent in in media from apoE^-/-^ B16-F10 cells (**Supplement Figure 2**). Multiplex results showed that secretion of the suppressive cytokine IL10 is diminished when DC were cultured in apoE^-/-^ CM compared to culture in WT CM. In a pro-inflammatory fashion IL1α, IL1β, RANTES, MIP1α, MIP1β, IL28 are all increased with apoE^-/-^ CM compared to WT CM (**supplement Figure 2**). To further delineate the DC suppressive effect directly, we tested the effect of the apoE peptide mimetic, COG133 on DC activation. In a dose dependent fashion, COG 133 induced secretion of the anti-inflammatory cytokine, IL-10 from activated DC. Furthermore, COG133 suppressed IL-1α, IL-1β and IL-23 in a dose dependent manner suggesting the immune-modulatory role of ApoE on DC function (**Supplement Figure 3**).

### ApoE peptide mimetic COG133 inhibits cytokine secretion induced by immunogenic tumor cells, while anti-APOE blocking antibody enhances cytokine secretion in tumor cell/splenocyte reactions

**Figure 3:**
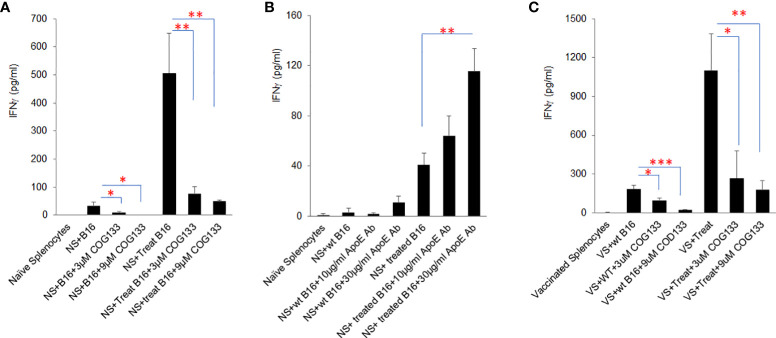
ApoE agonist peptide COG133 inhibits cytokine secretion induced by immunogenic tumor cells, while anti-APOE blocking antibody enhances cytokine secretion in tumor cell/splenocyte reactions. **(A)** Naïve and vaccinated splenocyte production of IFNγ was markedly reduced when splenocytes were co-cultured with immunogenic (BET/JQ1 treated) B16 tumor cells in the presence of the apoE agonist COG133. **(B)** Naïve splenocyte production of IFNγ was significantly increased when co-cultured with immunogenic B16 tumor cells with anti-APOE antibody. **(C)**. COG133 was also inhibitory of IFNγ production when vaccinated splenocytes were used for co-culture with treated immunogenic cancer cells. It is of interest to note that the levels of IFNγ production is significantly greater when vaccinated splenocytes were used for this experiment. NS: naïve splenocytes, splenocytes were collected from naïve C57BL/6 mice. Treated B16: B16 cells were expose to Myc inhibitor (0.25µM BET and 0.25µM JQ1) for 4 days, to induce immunogenicity. VS: vaccinated splenocytes. Irradiated 10^4^ WT B16 tumor cells and 100ug/ml anti-CTLA4 antibody were administered to C57BL/6 mice on day 0, and splenocytes were collected at day 7 after tumor cell inoculation. Data are representative of three independent experiments. Results are expressed as mean score ± SD. *p<0.05; **p<0.005; ***p<0.001, determined by unpaired two-tailed Student’s t-test.

To further investigate the influence of tumor secreted apoE on immune cell function, immunogenic B16 tumor cells (B16 cells treated with Myc inhibitors (0.25µM BET+0.25µM JQ1 for 4 days) were irradiated at 60 Gray and co-cultured with naïve C57/BL6 splenocytes in the presence of either apoE mimetic COG133 at 0.3, 3 and 9 µM or anti-APOE blocking antibody at 1, 10 and 30 µg/ml concentrations. Prior work from our laboratory has shown the immunogenic effect of treating cancer cells with Myc inhibitors and irradiation (19). IFNγ production was quantified by ELISA at 48h. Splenocytes produced high levels of IFNγ when co-cultured with Myc-inhibited immunogenic B16 cells as shown in **Figure 3**. Exposure of these cells to apoE mimetic COG133 repressed IFNγ production (6-fold reduction) (**Figure 3A**), while the presence of Anti-APOE antibody enhanced IFNγ production (3-fold increase) (**Figure 3B**). We also tested IFNγ production from vaccinated splenocytes following co-culture with treated and untreated B16 cells in the presence of COG 133. To obtain vaccinated splenocytes, 10^4 WT B16 tumor cells and 100µg/ml anti-CTLA4 antibody were administered to C57BL/6 mice on day 0, and splenocytes were collected at day 7 after tumor cell inoculation. Compared with naïve splenocytes, vaccinated splenocytes produced dramatically greater level of IFNγ, especially when they were co-cultured with Myc inhibited B16 tumor cells. This high level IFNγ was also suppressed by COG 133 at a dose dependent manor (**Figure 3C**). In addition to IFNγ, we also quantified other cytokine/chemokines using ProcartaPlex multiplex immunoassay. Fourteen out of 23 detectable cytokines were significantly upregulated when splenocytes were co-cultured with Myc-inhibited B16 tumor cells, including effectors: IFNγ, TNFα; stimulators, IL18, G-CSF, M-CSF; inflammatory factor: IL6; chemo attractive factors: CCL-5 (Rantes), CXCL-1, CCL-2, CCL-7, CXCL-2, CCL3; and regulatory factors: IL-10, IL6 (P < 0.05). Within these 14 cytokines, four of them including IFNγ, IL6, IL18 and RANTES (CCL5) were suppressed after exposure to apoE peptide mimetic COG133 in a dose dependent manner (**Supplement Figure 4**).

### The apoE receptors lrp8 and ldlr are dominantly expressed on activated T cells and dendritic cells and blocking lrp8 enhanced T-cell activation *in vitro*

**Figure 4:**
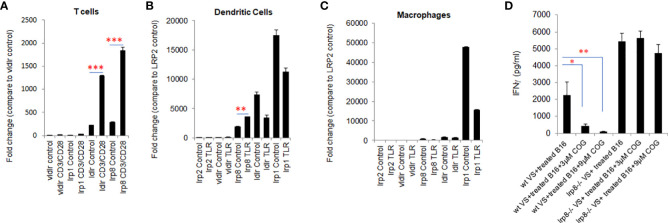
lrp8 is the most dominant receptor expressed on activated T cells and blocking lrp8 enhanced T-cell activation. **(A)** Expression of apoE receptors shows predominance of ldlr and lrp8 receptors on T cells, **(B)** lrp8, ldl and lrp1 receptors on dendritic cells, and **(C)** lrp1 receptors on macrophages as shown by quantitative real-time PCR (qPCR). Expression of ldlr and lrp8 was upregulated following T cell stimulation with CD3/CD28 beads. Expression of lrp8 was also upregulated following dendritic cell stimulation with TLR7/8. **(D)** The amount of IFNγ production from vaccinated splenocytes co-cultured with immunogenic WT B16 cells was quantified by ELISA assay. Results show that IFNγ production was significantly suppressed in the presence of the apoE agonist COG133, but suppression did not occur when splenocytes were harvested from lrp8^-/-^ mice. These findings suggest that T-cell function is at least partially inhibited by apoE through the lrp8 receptor pathway. Results are expressed as mean score ± SD. *p<0.05; **p<0.005; ***p<0.001, determined by unpaired two-tailed Student’s t-test.

The pattern of expression of apoE receptors on T cells and dendritic cells is not fully characterized. Here we examined the expression of apoE receptors on T cells, dendritic cells (DC) and macrophages using qPCR. All five of the receptor transcripts were shown to be expressed on T cells, DCs and macrophages. Ldlr and lrp8 were dominantly expressed on T cells (**Figure 4A**) whereas lrp1, lrp8 and ldlr are highly expressed on DC (**Figure 4B**). Lrp1 is highly expressed in macrophages (**Figure 4C**). Vldlr expression was relatively low, and lrp2 was barely detectable on these three cell types. We further examined the effect on expression of these receptors by stimulating T cells with CD3/CD28 beads and stimulating DCs and macrophages with a TLR7/8 agonist. Expression of lrp1, lrp8, ldlr and vldlr were all significantly upregulated on T cells following activation (**Figure 4A**), whereas only lrp2 and lrp8 were increased on DC after TLR stimulation (**Figure 4B**). This data together with previous studies (25, 26) suggest that ldlr and lrp8 (apoER2) are dominantly expressed and may be prominently engaged in apoE-mediated immune cell suppression.

To functionally define the role of the lrp8 receptor engagement in apoE suppression, we isolated vaccinated splenocytes from lrp8^-/-^ mice. These cells were co-cultured with BET/JQ1 treated (Myc-suppressed) immunogenic B16 cells for 48hr with or without exposure to the apoE mimetic COG133. ELISA results show that IFNγ production from splenocytes was inhibited by COG133 in WT mice, however the inhibitory effect was lacking in splenocytes from the lrp8^-/-^ mice (**Figure 4D**). In addition, using ProcartaPlex multiplex immunoassay, we also quantified other cytokines/chemokines produced in the reaction. Seventeen out of 27 detectable cytokines showed a similar pattern to IFNγ inhibition in WT mice that was reversed in lrp8^-/-^ splenocytes. These included effector function: IFNγ; stimulator function, IL18, GM-CSF, G-CSF; inflammatory cytokines: IL2, IL3, IL4, IL5, IL9, IL13, IL23, IL12p70; chemo attractant factors: MCP-1, MCP3, MIP-1α, CCL-5 (Rantes); and regulatory factors: IL6, IL-10 (P < 0.05). Representative data are shown in **Supplement Figure 5**. These findings suggest that T-cell function is at least partially inhibited by apoE through the lrp8 receptor pathway.

### Targeting apoE suppresses tumor growth with enhanced mouse survival in a murine melanoma model

**Figure 5:**
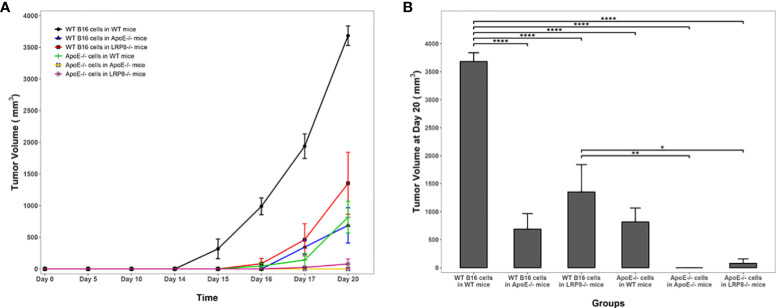
Knock out of apoE in both B16 tumor cells and in mice enhances host immunity and attenuates tumorigenicity. **(A)** In vivo, 104 WT or apoE-/- B16 cells were injected into the right leg of WT (n=9), apoE-/- (n=9) or lrp8-/- mice (n=9). The average tumor growth in each group (n=6) is compared. Tumor growth was significantly slower when apoE-/- B16 cells were injected into apoE-/- or lrp8-/- mice versus the other groups (two-way ANOVA; P <0.0001). **(B)** The final tumor volume between treatment groups at the end point of the experiment (Day 20) was also compared using a one-way ANOVA followed by Tukey HSD pairwise multiple comparisons between treatment groups. Tumor volumes at the end point of the experiment were significantly different between treatment groups (one-way ANOVA; P <0.0001). *p<0.05; **p<0.01; ****p<0.0001.

**Figure 6:**
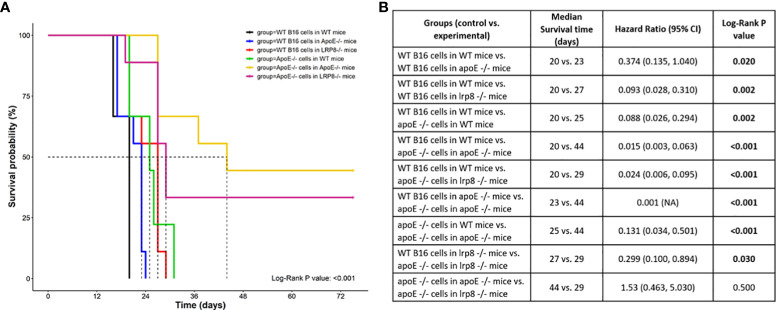
Survival curve **(A)** Survival was significantly better (n=9) when apoE^-/-^ B16 cells were injected into apoE^-/-^ or lrp8^-/-^ mice versus the other groups. **(B)** The median survival time and the cumulative survival probability were calculated and compared using the Kaplan-Meier survival estimator followed by a log-rank test, and the hazard ratio (HR) was calculated using the Cox proportional-hazards regression model. The comparison between the groups is shown in the table.

To additionally investigate the role of apoE on melanoma tumor growth *in vivo*, 1x10^4^ WT or apoE^-/-^ B16 cells were injected into the right flank of WT, apoE^-/-^ and lrp8^-/-^mice (n=9). The mice from each group were monitored for tumor growth and survival. The results show that targeting apoE in both the tumor cells and the host (apoE^-/-^ B16 cells injected into apoE^-/-^ C57/BL6 mice) results in delayed tumor growth (**Figure 5**), and significant rejection of tumor cell inoculation with improved overall survival (**Figure 6**). Tumor growth was also impaired when apoE^-/-^ B16 cells were injected into lrp8^-/-^ mice, suggesting apoE/LRP8 receptor engagement is important in mediating the apoE protective effect on tumor growth. Complete deletion of apoE in the tumor cells and in the host was most effective for inhibition of tumor growth, while apoE^-/-^ B16 cells injected into WT mice, or WT B16 cells injected into apoE^-/-^ mice or lrp8^-/-^ mice partially suppressed tumor growth compared to control. The suppressive effect observed on tumor growth *in vivo* with apoE suppression appears indirect as apoE KO cells proliferate normally.

### The combination of apoE^-/-^ tumor cells administered to apoE^-/-^ mice resulted in the most profound activation of immune pathway signaling and cell infiltrates in the tumor microenvironment

**Figure 7:**
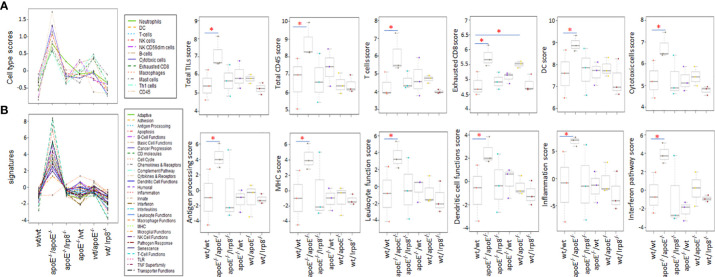
The first three tumors that reached 15mm in each of the experimental groups were harvested and the immune cell infiltrate and immune pathway activation was analyzed with nanostring Pancancer immune profiling. **(A)** Immune cell type scores and **(B)** immune pathway (signature) scores show that immune cell infiltrates and immune pathway activation were greatest when apoE-/- cells were injected into apoE-/- mice. Dot line plots show the score trends of 12 immune cell lines and 29 immune pathways. Box plots show selective representative score comparisons. Results are expressed as mean score ±SD. *p<0.05, determined by unpaired two-tailed Student’s t-test. Wt/wt: wt B16 cells injected in wt mice; apoE-/-/apoE-/-: apoE-/- cells injected in apoE-/- mice; apoE-/-/lrp8-/-: apoE-/- cells injected in lrp8-/- mice; apoE-/-/wt: apoE-/- cells injected in wt mice; wt/apoE-/-: wt cells injected in apoE-/- mice; wt//lrp8-/-: wt cells injected in lrp8-/- mice.

**Figure 8:**
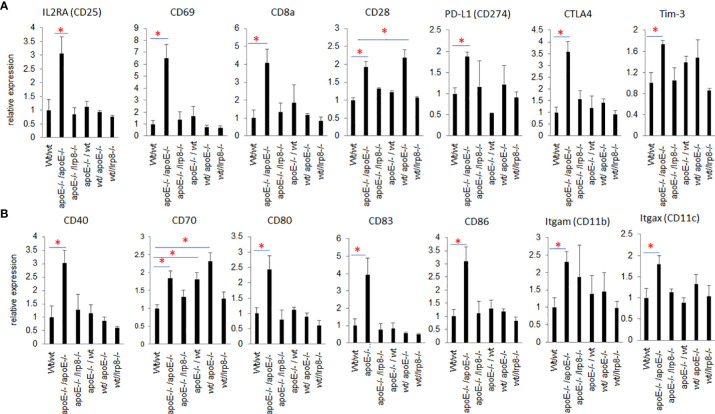
Nanostring PanCancer Immune Profiling analysis of RNAseq of activation markers for T cells and DCs that infiltrated into the tumors from the 6 different groups. **(A)** Activated T cell genes and **(B)** activated DC genes were all statistically significantly increased in the tumor from apoE-/-/apoE-/- group when compared with wt/wt control groups. The relative gene expression level was indicated on the y-axis and tumor groups are listed along the x-axis. Results are expressed as mean score ±SE. *p<0.05, determined by unpaired two-tailed Student’s t-test.

To evaluate if the protective effect against tumor growth with apoE targeting is immune mediated, we harvested the first 3 mice from each group that grew tumors to 15mm for tumor immune profiling. Nanostring analysis of immune cell infiltrates and activation of immune signaling pathways revealed that the apoE**^-/-^** mice in which apoE**^-/-^** cells were inoculated, demonstrated the greatest number of immune cell infiltrates (**Figure 7A**) as well as the highest activation of immune signaling pathways (**Figure 7B**) based on the expression of signature marker gene transcripts when compared to the other groups. Wild type tumors in apoE**^-/-^** mice or WT mice receiving the apoE**^-/-^** cells demonstrated more activation of immune pathways and cellular infiltrates as determined by RNA transcripts than WT controls, but these effects were only partial and inferior to the immunity induced when apoE was abolished in the system (apoE^-/-^ cells in apoE^-/-^ B57/BL6 mice). The expression level of the gene transcripts of multiple activation markers for T cells (**Figure 8A**) and dendritic cells (DCs) (**Figure 8B**) were also compared within 6 tumor groups. Results showed that the markers for activation T cells including interleukin-2 receptor alpha chain (IL2RA, or CD25), CD69, CD8a, CD28, check point inhibitors PD-L1 and CTLA4, and T cell exhaustion marker Tim-3 were all significantly enhanced in the tumor from the apoE**^-/-^** mice in which apoE**^-/-^** cells were inoculated (apoE**^-/-^**/apoE**^-/-^**) compared with control group (wt/wt), PD1 and Lag 3 were slightly increased, but they were not statistically significant (data not shown). CD28 was also upregulated in the tumor from the apoE**^-/-^** mice in which wt tumor cells were inoculated (wt/apoE**^-/-^**) (**Figure 8A**). For DCs, the activation genes including CD40, CD70, CD80, CD83, CD86, CD11b and CD11c were all significantly increased in

**Figure 9:**
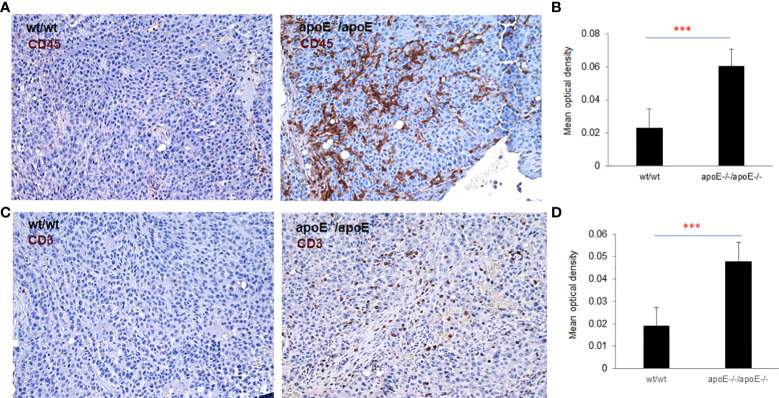
To validate nanostring results, both CD45 and CD3 expression were examined with IHC staining in the tumors from WT or apoE-/- mice following injection with WT or apoE-/- tumor cells. Representative images of CD45 **(A)** and CD3 **(C)** staining visualized with DAB (brown) and counterstained with hematoxylin (blue, nuclei). **(B, D)** Optical density (mean gray value) obtained by color deconvolution analysis. Optical density graph bars represent the mean ± SD (n = 30 images). ***p<0.001, determined by unpaired two-tailed Student’s t-test. Wt/wt: wt B16 cells injected in wt mice; apoE-/- /apoE-/-: apoE-/- cells injected in apoE-/- mice.

apoE**^-/-^**/apoE^-/-^ group compared with controls. CD70 was also increased in wt/apoE**^-/-^** and apoE**^-/-^**/wt groups. (**Figure 8B**). These observations show that apoE secreted from the tumor or produced in the host impair immunity and establish the potent role that apoE plays in suppressing tumor immunity in the mouse melanoma model. Surprisingly, there was no upregulation of immune pathway scores nor enhanced immune cells scores in lrp8**^-/-^** mice. These observations do not correlate with *in vitro* findings nor *in vivo* growth rates, but this may have been specific to the three mice sampled that developed tumors early in this group. To validate nanostring RNA transcript results in the apoE targeted group, we performed immunohistochemistry staining of the immune cell marker CD45 (lymphocyte common antigen) and CD3 (T cell marker) on the same tumor samples that we used for nanostring analysis. Results showed that the apoE**^-/-^** mice in which apoE**^-/-^** cells were inoculated, have significantly more CD45 (**Figures 9A, B**) and CD3 (**Figures 9C, D**) positive immune cell infiltrates than WT control. The other groups were not studied as these gross observations do not have the same objectivity or power of analysis as the nanostring assay.

### ApoE knock out in B16 tumor cells induces potent immunogenicity

**Figure 10:**
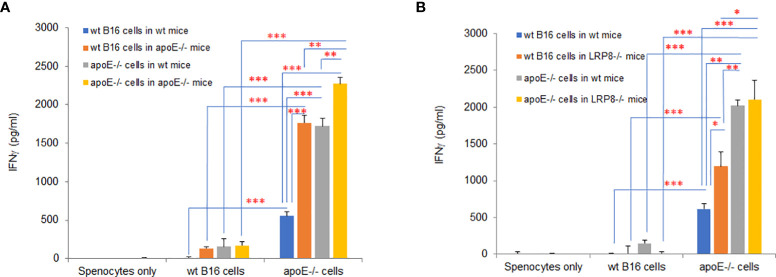
ApoE knock out in B16 tumor cells induces potent immunogenicity. Wild type and apoE^-/-^ mice **(A)** or wild type and lrp8^-/-^ mice **(B)**, were inoculated with either WT B16 or apoE^-/-^ cells, with anti-CTLA4 antibody on day 0. Splenocytes were harvested on day 7 and co-cultured with either WT B16 or apoE^-/-^ B16 cells for 48hr, following which IFNγ levels in media were compared with ELISA assay. Results show that IFNγ production is highest for all groups when apoE^-/-^ cells are used. These findings re-iterate the potent inhibitory effect of apoE on immune cell activation in the cancer environment. Results are expressed as mean score ± SD. *p<0.05; **p<0.005; ***p<0.001, determined by unpaired two-tailed Student’s t-test.

In the mouse model, apoE appears to be an immune modulator critical for enabling tumor cell growth through suppression of immune activation. To determine if knocking out apoE in tumor cells induced immunogenicity, we vaccinated wild type and apoE^-/-^ mice (**Figure 10A**) or wild type and lrp8^-/-^ mice (**Figure 10B**) with WT B16 or apoE^-/-^ B16 tumor cells and CTLA4 Ab and then collected splenocytes from these vaccinated mice 6 days later. We then co-cultured the splenocytes with WT B16 or apoE^-/-^ B16 cells to evaluate IFNγ secretion as a marker of induced immunity. ApoE^-/-^ B16 cells induced robust immunity in whatever circumstance they were tested either as the primary immunogen or with re-stimulation of splenocytes (**Figures 10A,B**). These findings re-iterate the potent inhibitory effect of apoE on immune cell activation and present an opportunity to exploit this pathway to enable tumor immunity and cancer immunotherapy.

### ApoE RNA-seq expression is abundant in cutaneous melanoma but is not associated with PD1, PD-L1 or immune cell infiltrate RNA-seq expression

**Figure 11:**
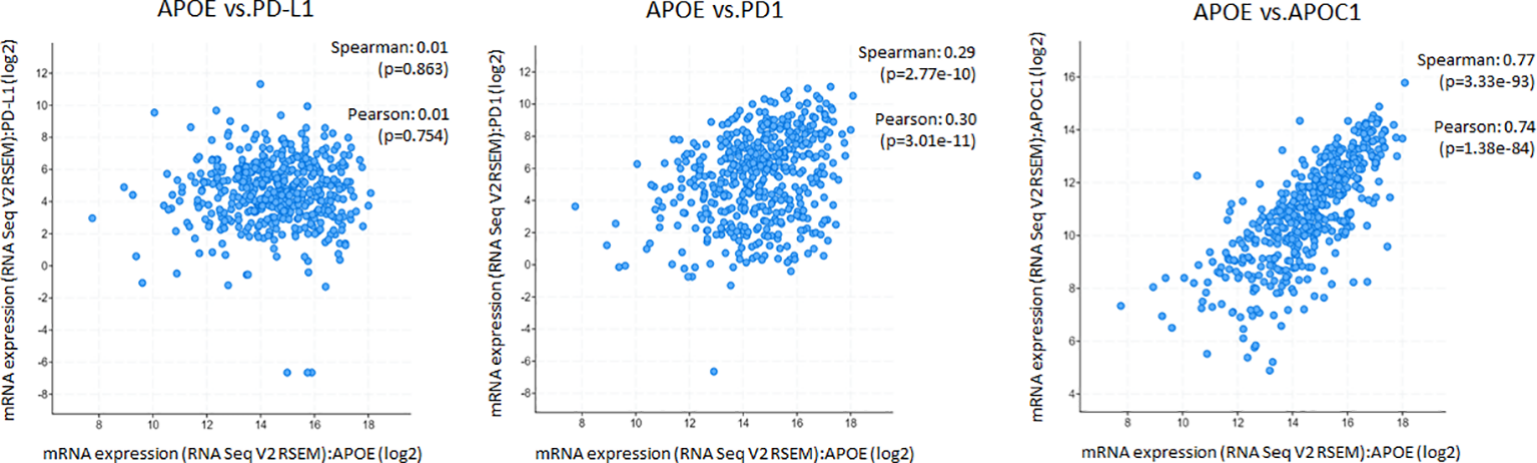
ApoE RNA-seq expression is abundant in cutaneous melanoma but is not associated with PD-L1 or PD1. Scatterplots showing mRNA expression correlation of APOE (x-axis) with PD-L1, PD1 and APOC1 from the TCGA melanoma datasets based on their RNA-seq gene expression values (measured by RSEM algorithm). APOE expression did not correlate with PD-L1 or PD1 but did positively correlate with APOC1 expression which was used as a control gene. The correlation was evaluated by the Spearman correlation coefficient with a cut-off value of 0.5 and P-value used a cut-off value of 0.05.

**Figure 12:**
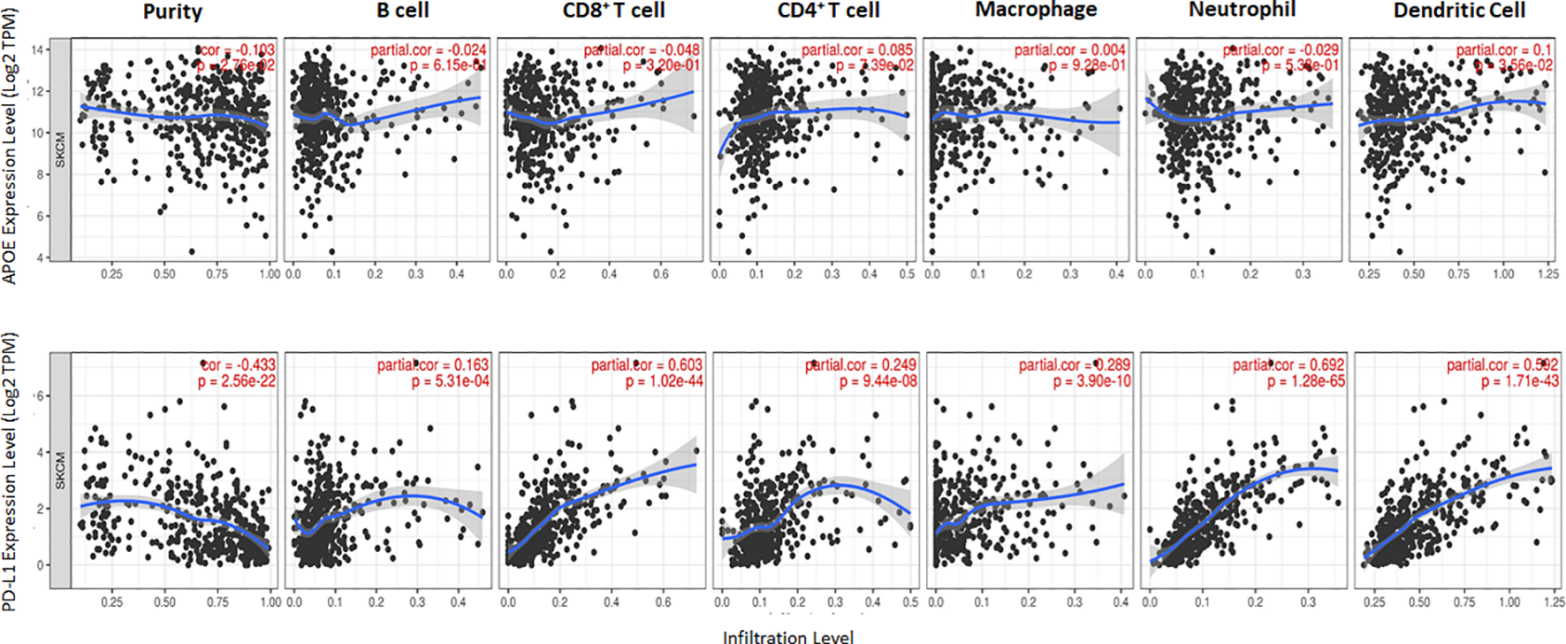
Scatterplots show the tumor purity-corrected partial Spearman’s rho value and the correlation between gene expression with infiltration of six immune cell estimates. Top row is APOE expression while the bottom row depicts PD-L1. PD-L1 correlated with CD8 T cell and neutrophil infiltrates, while APOE does not correlate with immune cell infiltrates. The correlation was evaluated by the Spearman correlation coefficient with a cut-off value of 0.5 and P-value used a cut-off value of 0.05.

**Figure 13:**
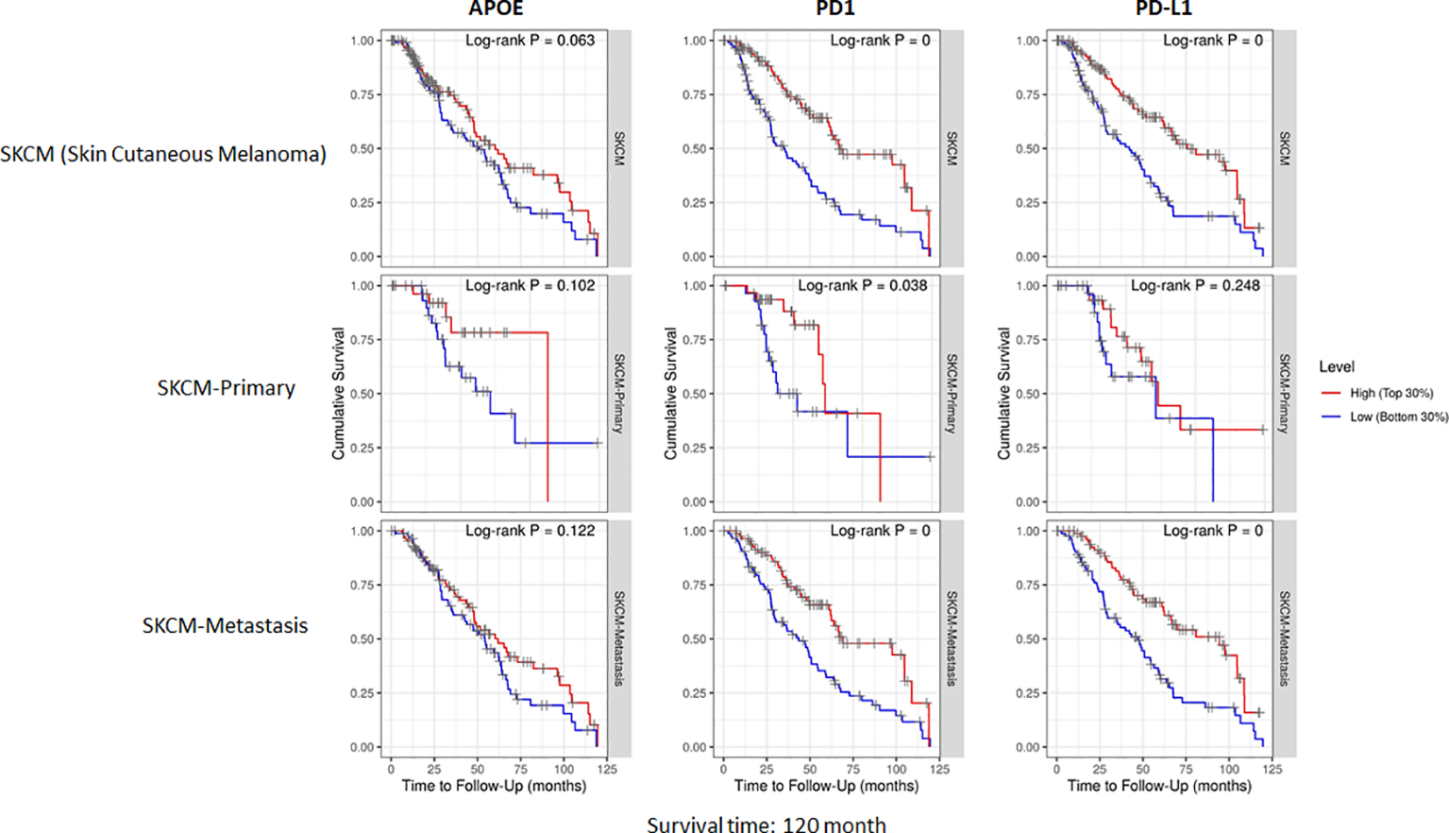
Cumulative survival of patients associated with bifurcate gene expression at 30%. TCGA database includes primary and metastasis samples (n=462). APOE expression did not associate with survival, while PD-L1 and PD1 was associated with survival at this level of analysis. The correlation was evaluated by the Spearman correlation coefficient with a cut-off value of 0.5 and P-value used a cut-off value of 0.05.

The potent immune-suppressive effects of ApoE in the melanoma mouse model suggests that APOE may be an important regulator of immunity in human melanoma. To evaluate its association with human melanoma, we analyzed TCGA melanoma datasets based on RNA-seq gene expression values (measured by RSEM algorithm) in 462 patient tumors. We evaluated expression and correlation with other checkpoints (**Figure 11**), immune cell infiltrates (**Figure 12**) and patient survival (**Figure 13**). APOE is abundantly present, however, it did not correlate with PD-L1 or PD1 expression, two checkpoints expressed on tumors, but did positively correlate with APOC1 expression APOC1 was used as a positive control in this analysis (**Figure 11**). APOE did not correlate with RNA-seq gene expression of T-cell, neutrophil and dendritic cells infiltrates whereas PD-L1 expression correlated with the presence of these three cell phenotypes (**Figure 12**). Also, APOE did not correlate with survival at a 30% bifurcate gene analysis, whereas the expression of PD-L1 and PD1 both positively correlated with survival of cutaneous melanoma (**Figure 13**). The high expression of checkpoints like PD-L1 and PD1 associated with cell infiltrates and survival curves, suggest that these genes are expressed in inflammatory tumors that have a better prognosis. APOE expression however appears to be independent of inflammatory phenotype in these tumors and may act as a separate and independent pathway in suppressing anti-tumor T-cell immunity.

## Discussion

In prior published work, we showed a distinctly different response to the same tumor vaccine protocol in a mouse neuroblastoma and melanoma tumor model (19). The neuroblastoma mouse model was remarkably sensitive to tumor vaccination even at a high dose of tumor cell inoculation whereas a low dose melanoma model was surprisingly resistant. This differential response could imply that intrinsic tumor cell characteristics and/or differences in tumor/host immunity are present that may account for differences in immune resistance.

In the current study we assess immunosuppressive modulators in the melanoma model and the modulator that was most highly expressed by qPCR in the B16-F10 melanoma is ApoE. We profiled over 700 immune associated gene transcripts in the melanoma B16-F10 cells and identified 18 genes with known immunosuppressive characteristics that were detected. ApoE was notably detected and found to be more abundant than any of the other known immunosuppressive transcripts identified. Besides its role in cholesterol transport, Apolipoprotein E (apoE) has considerable immunomodulatory properties (4, 18, 27–29). ApoE is shown to suppress lymphocyte proliferation (30) and modulate immune activation by acting on antigen-presenting cells (2, 30), implicating apoE as a suppressant of immune function. We found ApoE to be abundantly expressed in the B16-F10 melanoma cell line and was actively secreted by these tumor cells into the serum of the host as the tumors establish and grow. These findings raised suspicion that apoE contributes to immune escape.

Both the expression of apoE as well as the secretion of apoE from the tumor cells seems to have clear immunosuppressive effects. Our studies show that tumor cells depleted of apoE can stimulate remarkable immune activation in co-culture experiments. The conditioned media from apoE^-/-^ tumor melanoma cells alone did not suppress T-cell function like that of WT tumor cell conditioned media. To understand the mechanism by which apoE functions we investigated specific effects of the apoE peptide mimetic COG133 on T-cell and DC function. The suppressive effect appears to be evident on both stimulated DCs and T-cells as determined by cytokine secretion. We identified the dominant receptors on these cells showing that of the low-density lipoprotein (ldlr) receptor family evaluated, lrp8 and ldlr were most abundant and responsive to stimulation in T-cells while ldlr and lrp1 were present in DCs. In co-culture experiments, the suppressive effect of apoE on vaccinated splenocytes was absent when the splenocytes were harvested from lrp8^-/-^ mice. These findings suggest that T-cell function is at least partially dependent on apoE-lrp8 receptor engagement.

To investigate the premise of ApoE being a potent immune checkpoint, we challenged WT, apoE^-/-^ and lrp8^-/-^ mice with WT and ApoE knock out B16-F10 melanoma tumor cells. Results show remarkable suppression of tumor growth when ApoE is absent from the system with significant tumor elimination when compared with WT controls. ApoE in tumor cells or endogenously produced in the mice was capable of suppressing tumor immunity and significant tumor immunity only occurred when ApoE was absent from both tumor cells and the murine host. Immuno-phenotyping of the tumor micro-environment in mice that grew tumors, revealed the highest levels of immune pathway activation and immune cell infiltrates in the apoE^-/-^ mice that received apoE^-/-^ B16 melanoma cells as detected by RNA transcripts. The effect of apoE seems to be at least partially mediated by its interaction with the Lrp8 receptor as this group also had slower tumor growth and rejection of tumor cells when apoE^-/-^ melanoma cells were inoculated into Lrp8^-/-^ mice, endorsing the *in vitro* findings in splenocyte studies. However, the immune mediated effect was not as apparent in the lrp8^-/-^ mice, as activation of immune pathway and cellular RNA transcripts were not universally increased liked that observed in the complete apoE knock out tumor/mouse model. These findings may be limited and underestimate the ultimate tumor immunity as the tumor micro-environment was analyzed from the first three mice in each group that developed tumor and do not necessarily represent the mice with delayed or absent tumor growth.

Several potential shortfalls of our findings are evident considering prior literatureThe lack of myeloid-derived suppressor cells (MDSC) analysis in the B16-F10 mice models used is evident. Previous studies have shown that the liver-X nuclear receptor (LXR)/ApoE axis reduce MDSC abundance in murine melanoma models by binding to Lrp8 receptor on MDSC. MDSC blockade by LXR-induced ApoE enhanced cytotoxic T cell activation in B16-F10 bearing mice and patients, eventually leading to reduced melanoma growth (18). Hence, a further analysis of the innate and adaptive immune responses (including possibly depletion strategies to validate the model) is required to fully understand the implications of apoE in tumor immunity. Another potential limitation of our study is the assumption of cytokine responses observed. Cytokines have complex roles in immune response modulation, and it is also shown that IL-10 is considered to be immune-stimulatory as opposed to immune-suppressive (31) while IL-1a/b can be immune-suppressive. Thus, the results described for ApoE secreted from B16 melanoma tumor cells may impair activation of pro-inflammatory DCs and can be interpreted in an opposite direction. Our results also add to a conflicted literature in which apoE can mediate either pro- or anti-tumor effects. We employ CRISPR gene editing to assess the tumor-intrinsic effects of ApoE. Previous studies have indicated that regardless of the gene being targeted, that CRISPR/Cas9 manipulation can yield tumor cells of increased immunogenicity based on expression of Cas9-derived xenoantigens (32). Thus, reduced growth rate and increased immunogenicity of ApoE-/- B16 melanoma cells *in vivo* could be more dependent on Cas9 than ApoE-deficiency, however several of our B16-F10 melanoma controls do have CRISPR/Cas9 expression alone that do not seem to alter immunogenicity.

Despite these contradictions, our findings suggest that apoE plays a substantial immunomodulatory role with multiple inhibitory effects on T cell function, inflammatory cytokine response, and activation of dendritic cells. ApoE also protects tumors by suppressing tumor cell immunogenicity. ApoE^-/-^ B16 cells induced robust immunity in whatever circumstance they were tested either as the primary immunogen *in vivo* or with re-stimulation of splenocytes ex vivo. Together, the multiple experiments presented, establish the potent inhibitory effects of apoE on immune cell function and activation of immune signaling in the melanoma mouse model. These findings present an opportunity to exploit this pathway for enabling tumor immunity and cancer immunotherapy.

To understand the correlation between immunity and APOE expression in patients with melanoma, we accessed the raw RNA-seq data from the TCGA melanoma datasets (measured by RSEM algorithm) (n=462). Although APOE was abundantly present in the tumors, there was no correlation between APOE and PD1 or PD-L1 expression and there was also no association between APOE and cell infiltrates as there was between PD-L1 and T-cell/neutrophil expression. The findings do not take into consideration the serum levels of ApoE in the patients themselves. The observations from our mouse model imply that the absence of tumor ApoE alone is not sufficient to induce immunity in that the serum level of the host also has significant suppressive effects on induced immunity. In our patient analysis, we did not find any association with APOE and inflammatory tumors as we did for PD1 and PD-L1, nor did we find any association with patient survival. ApoE seems to be a potent suppressor of immunity and therefore may act independently of the classic well described checkpoints.

In summary, apoE plays a broad role in immune resistance observed in the WT B16 melanoma tumors. ApoE inhibits activation of immune cells, inflammatory signals, and tumor immunogenicity both locally and systemically *via* cellular secretion and host production. This work shows that tumor immunity can be restored and enhanced by targeting apoE. These findings identify apoE as a novel tumor checkpoint and an obvious target for improving tumor immunity with cancer immunotherapy.

## Data availability statement

The datasets presented in this study can be found in online repositories. The names of the repository/repositories and accession number(s) can be found in the article/**Supplementary Material**.

## Ethics statement

The animal study was reviewed and approved by The Institutional Animal Care and Use Committee (IACUC) of Children’s National Hospital, Washington, DC.

## Author contributions

AS conceived the idea and acquired funding for the study. AS, XW and MS wrote the original draft, and all authors reviewed and edited the manuscript; XW, MBa, PS, BT, AJ, MBe, and AS interpreted the data, made the figures, planned the experiments, performed and analyzed the experiments. All authors reviewed, edited, and approved the final manuscript.

## Funding

This work has been supported in part by the EVAN Foundation, the Catherine Blair foundation, and the Michael Sandler Research Fund as well as the Sheikh Zayed Institute for Pediatric Surgical Innovation. Author receiving: AS.

## Acknowledgments

We thank Mrs Karuna Panchapakesan and Dr Susan Knoblach from the Children’s National Genetic and Bioinformatics Core for helping with Nanostring experimental setup, and Dr. Christopher Lazarski for his assistance in flow cytometry experimental setup and data analysis.

## Conflict of interest

Authors BT, AJ, and MBe were employed by Integrated DNA Technologies, Inc.

The remaining authors declare that the research was conducted in the absence of any commercial or financial relationships that could be construed as a potential conflict of interest.

## Publisher’s note

All claims expressed in this article are solely those of the authors and do not necessarily represent those of their affiliated organizations, or those of the publisher, the editors and the reviewers. Any product that may be evaluated in this article, or claim that may be made by its manufacturer, is not guaranteed or endorsed by the publisher.
