# Supplementary material for: Tumor Apolipoprotein E is a key checkpoint blocking anti-tumor immunity in mouse melanoma
Source: Front Immunol. 2022 Oct 19;13:991790. doi: 10.3389/fimmu.2022.991790 (PMC9626815; doi:10.3389/fimmu.2022.991790)

Supplement Figure 1

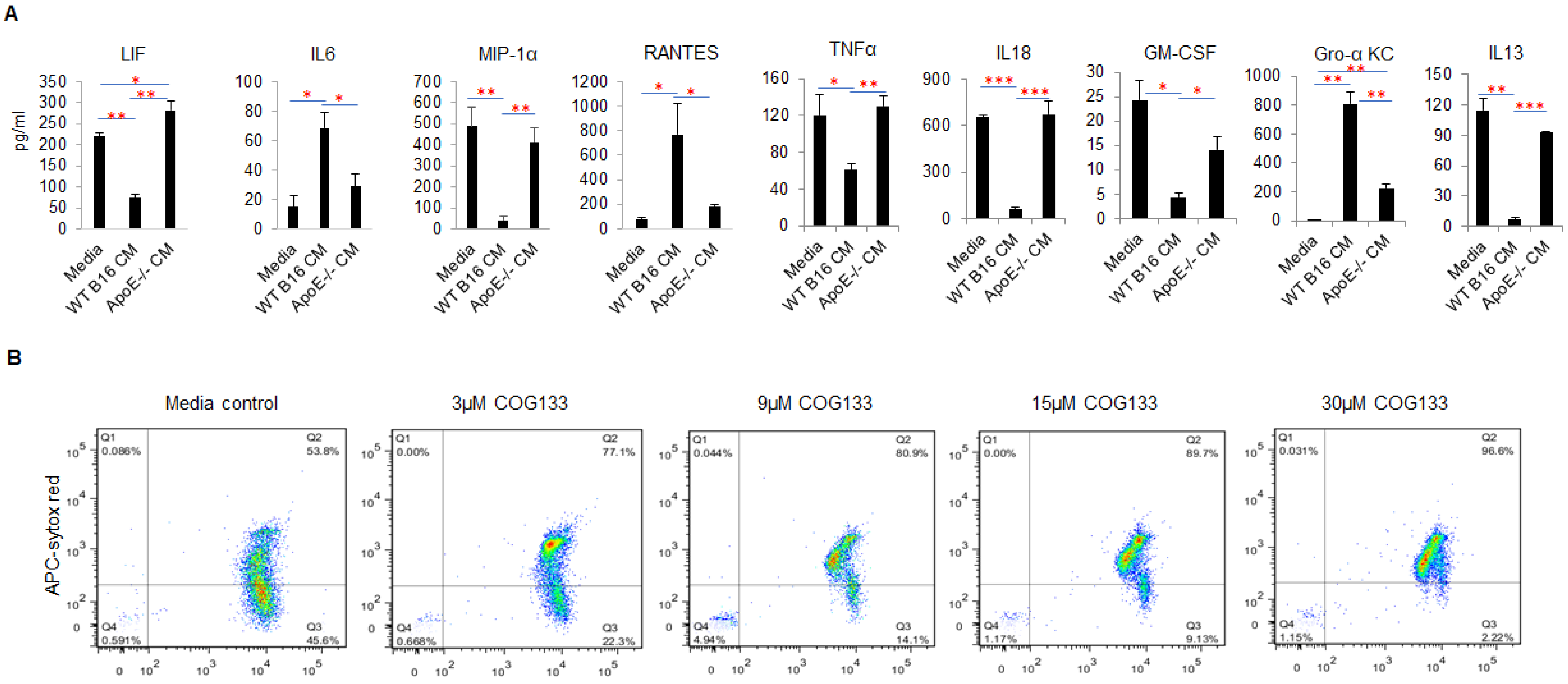

Supplement Figure 2

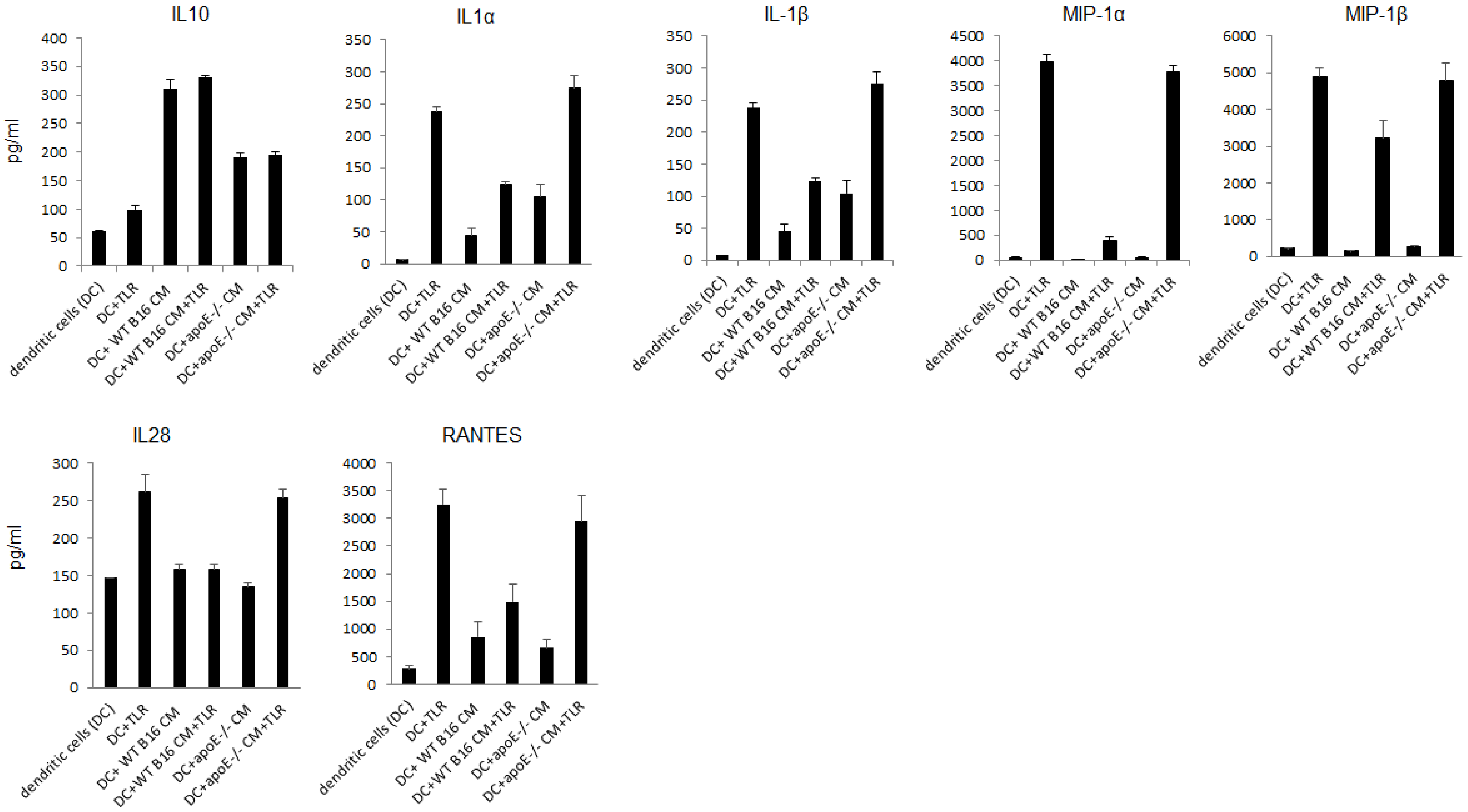

Supplement Figure 3

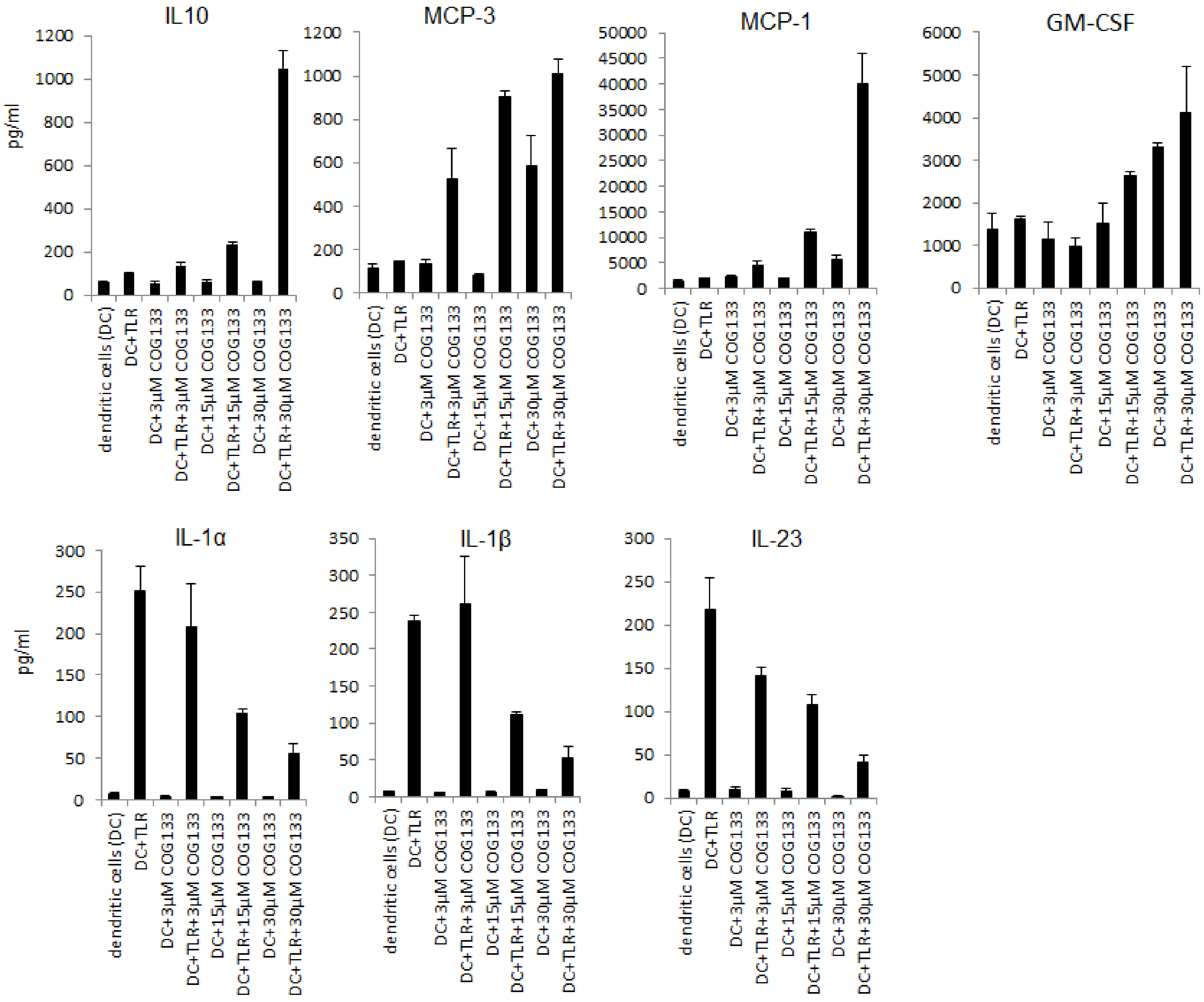

Supplement Figure 4

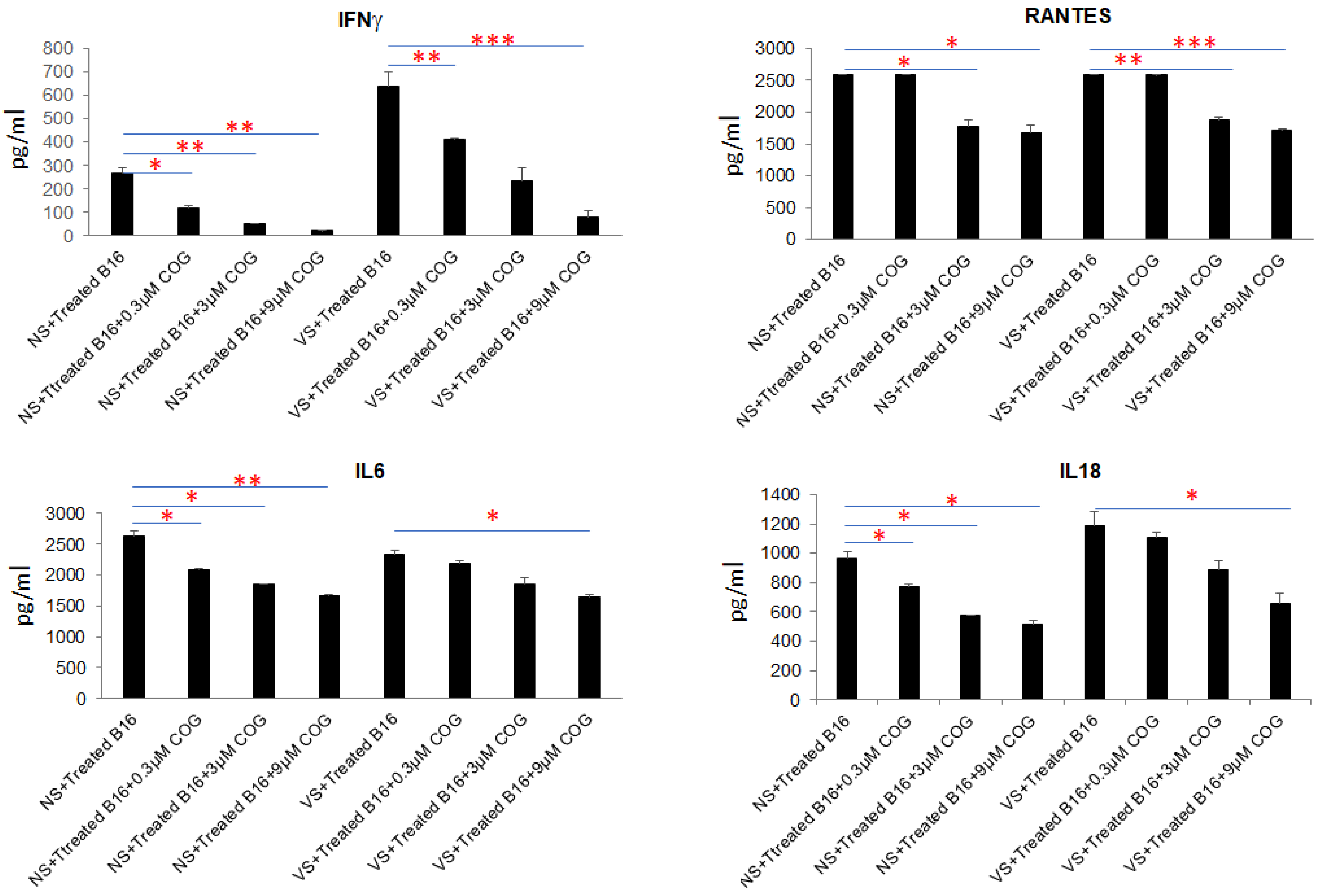

Supplement Figure 5

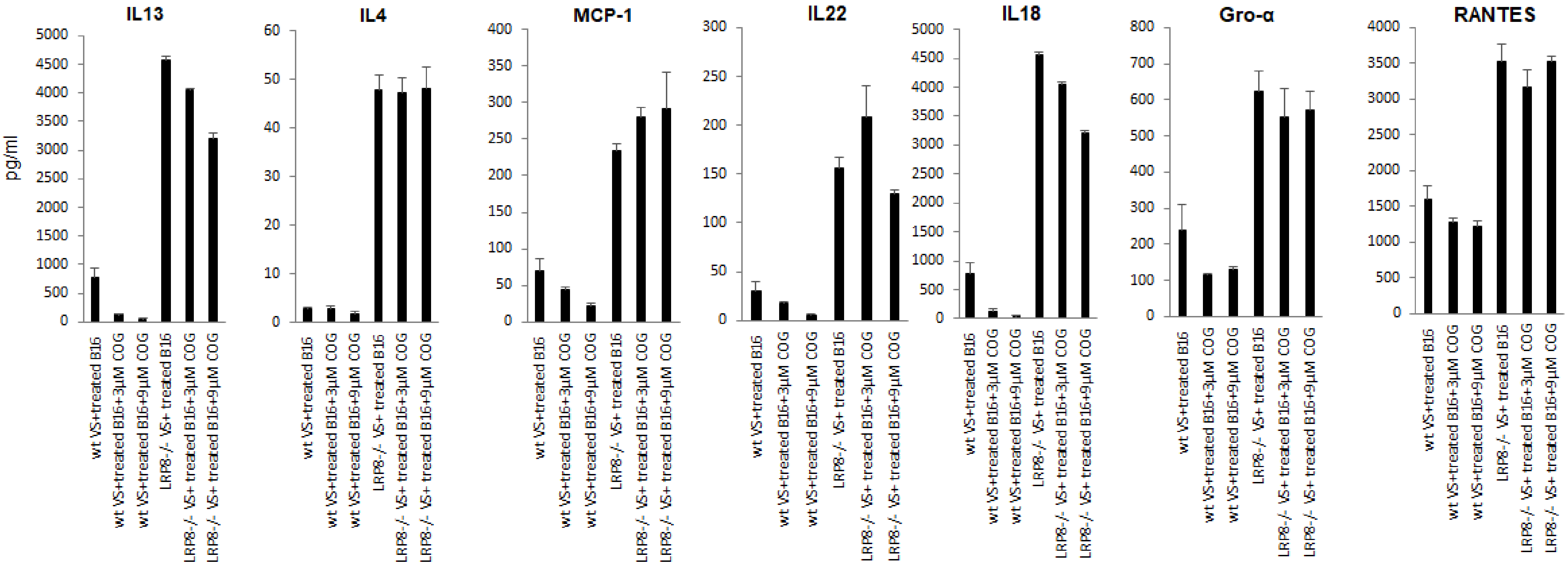

Supplement: Supplementary file 1 [file DataSheet_1.pdf]
