# Supplementary material for: Tumor Apolipoprotein E is a key checkpoint blocking anti-tumor immunity in mouse melanoma
Source: Front Immunol. 2022 Oct 19;13:991790. doi: 10.3389/fimmu.2022.991790 (PMC9626815; doi:10.3389/fimmu.2022.991790)
Supplement: Supplementary file 2 [file DataSheet_2.docx]

**Supplementary Table 1. A list of names and targeting sequences of crRNAs used in screening for Mm apoE knock out.**

| **crRNA name** | **Sequence** |
| --- | --- |
| Mm.Cas9.APOE.1-A | GAGGATCTACGCAACCGACT |
| Mm.Cas9.APOE.1-B | CAACGAGGTGCACACCATGC |
| Mm.Cas9.APOE.1-C | GAGGTGACAGATCAGCTCGA |
| Mm.Cas9.APOE.1-D | GACGCTGTCTGACCAGGTCC |
| Mm.Cas9.APOE.1-E | GGACACTATGACGGAAGTAA |
| Mm.Cas9.APOE.1-F | CTGGTGGAGCAAGGTCGCCA |
| Mm.Cas9.APOE.1-F | CTGGTGGAGCAAGGTCGCCA |

**Supplementary Table 2. A list of primers used for NGS of target regions in Mm ApoE to evaluate total editing and INDEL profile. Uppercase letters indicate target-specific annealing sequence in PCR1, and lowercase letters indicate sequence for incorporating P5 and P7 Illumina adapters to amplicon ends in PCR2.**

| **Primer name** | **Sequence** |
| --- | --- |
| NGS For 1 | acactctttccctacacgacgctcttccgatctAGACCCAAAAAGACTGTAGG |
| NGS Rev 1 | gtgactggagttcagacgtgtgctcttccgatctTGCCGAGGGTGAAAGAGCTG |
| NGS For 2 | acactctttccctacacgacgctcttccgatctGCCTTCATCTCCTTCCTGTG |
| NGS Rev 2 | gtgactggagttcagacgtgtgctcttccgatctCCTCTGTGCTCTGGCCCAGC |
| NGS For 3 | acactctttccctacacgacgctcttccgatctAGGCTGGGCAAAGAGGTGCA |
| NGS Rev 3 | gtgactggagttcagacgtgtgctcttccgatctCGCTTCTGCAGATCCTCGGC |
| NGS For 4 | acactctttccctacacgacgctcttccgatctTGCCGAGGATCTGCAGAAGC |
| NGS Rev 4 | gtgactggagttcagacgtgtgctcttccgatctGCCGCCCTCGGATGCGGTCA |

**Supplemental Figure legends:**

**Supplement Figure 1. ApoE has a repressive effect on T cell function and viability.** **(A)** T cells isolated from naïve mouse spleens were cultured in RPMI media, WT B16 conditioned media and apoE^-/-^ B16 conditioned media for 48hr and the media was analyzed with ProcartaPlex multiplex immunoassay. Results show the production of pro-inflammatory cytokines and chemokines such as LIF, MIP-1α, TNFα, IL18, GM-CSF and IL-13 were suppressed while production of IL-6, RANTES and Gro-α KC were enhanced when T cells were cultured in WT B16 conditioned media. Remarkably, these effects were reversed by incubating T-cells with conditioned media from apoE^-/-^ cells, similar to T-cells activated in control RPMI media alone. Results are expressed as mean score ±SD. *p<0.05; **p<0.005; ***p<0.001, determined by unpaired two-tailed Student’s t-test. **(B)** The effect of apoE agonist peptide COG133 on the viability of activated mouse T cells was tested by culturing the cells in the presence of the indicated concentrations of peptide for 48hr and cell death was quantified with flow cytometry using the APC-conjugated Sytox Red dead cell stain. T-cell viability decreases in a dose dependent fashion in the presence of COG133 ApoE agoist. The number in Quadrant 2 is the percentage of dead cells.

**Supplement Figure 2. Role of apoE on effector function of dendritic cells.** Mouse primary bone-marrow derived dendritic cells (DC) were cultured in the presence of conditioned medium (CM) from WT B16 and apoE^-/-^ cells for 48hr with or without toll like receptor (TLR7/8) stimulation. Multiplex ELISA assay was used to detect cytokines and chemokines and results showed that WT B16 CM enhances the production of anti-inflammatory cytokine IL-10 while downregulating the production of proinflammatory cytokines IL1α, IL1β, MIP-1α and MIP-1β, IL28 and RANTES. This effect was reversed when DCs were cultured in apoE^-/-^ cell CM.

**Supplement Figure 3.** **Dendritic cell function is modulated by apoE agonist COG133.**

Mouse bone-marrow derived dendritic cells (DC) were cultured in the presence of the indicated concentrations of apoE agonist COG133 for 48hr. COG133 increased the production of anti-inflammatory IL-10, GM-CSF, and chemokines MCP-1 and MCP-3 by TLR7/8 activated DC while decreasing the levels of proinflammatory cytokines IL-1α, IL-1β and IL-23 in a dose-dependent manner as determined by multiplex ELISA.

**Supplement Figure 4.** **Vaccination with immunogenic WT B16 tumor cells enhances splenocyte response which is dampened by the presence of apoE agonist COG 133.** Splenocytes from naïve mice (NS) as well as mice vaccinated with 10^4^ WT B16 and 100µg/ml anti-CTLA4 antibody (VS) were co-cultured for 48 hr with either WT B16 cells or Myc-inhibited immunogenic B16 tumor cells in the presence of the indicated concentrations of apoE agonist COG133. Multiplex ELISA shows the suppressive effect of the apoE agonist in production of IFNγ, IL-6 and IL-18 in the cocultures with naïve splenocytes and this suppressive effect although diminished in vaccinated splenocytes is still observed.

**Supplement Figure 5.** **ApoE affects T cell function at least partially through lrp8 receptor pathway.** Splenocytes were isolated from vaccinated WT mice as well as lrp8^-/-^ mice and cocultured for 48 hr with Myc-inhibited immunogenic B16 cells, in the presence of the indicated concentrations of apoE agonist COG133. Multiplex immunoassay shows a suppression of proinflammatory cytokines and chemokines IL-13, IL-4, IL22, IL18, MCP-1, RANTES and Gro-α in the cocultures with WT vaccinated splenocytes. However, this suppressive effect is diminished in lrp8^-/-^ vaccinated splenocytes.
